# Blood, sweat, tears and fibrosis: when overtraining injures the liver

**DOI:** 10.20517/evcna.2025.56

**Published:** 2025-11-10

**Authors:** Enis Kostallari

**Affiliations:** ^1^Division of Gastroenterology and Hepatology, Mayo Clinic, Rochester, MN 55905, USA.; ^2^Department of Biochemistry and Molecular Biology, Mayo Clinic, Rochester, MN 55905, USA.

**Keywords:** Liver fibrosis, skeletal muscle liver axis, extracellular vesicles, lactate, condensates

## Abstract

The crosstalk between the skeletal muscles and the liver is receiving growing attention, as patients with chronic liver disease often develop a loss of skeletal muscle mass. In these patients, particularly those with metabolic dysfunction-associated steatotic liver disease, physical exercise improves insulin sensitivity and hepatic steatosis. However, excessive exercise may impair mitochondrial function, inflammation, and liver health. The study by Liu *et al*. demonstrates that overtraining promotes liver fibrosis through myocyte-derived small extracellular vesicles. Here, we comment on the novelty of these findings and areas to be developed in the future.

The crosstalk between the skeletal muscles and the liver is receiving growing attention, as patients with chronic liver disease often develop sarcopenia^[1]^. Sarcopenia, or the progressive loss of skeletal muscle mass, strength, and function, affects up to 70% of patients with liver cirrhosis^[1]^ and 50% of those undergoing liver transplantation^[2]^. Sarcopenia is associated with altered quality of life, increased mortality, and post-transplant complications^[3]^, and it can persist even after liver transplant^[4]^. This underlines the existence of an important skeletal muscle-liver axis. Exercising increases muscle mass and mitigates sarcopenia. In patients with chronic liver disease, particularly those with metabolic dysfunction-associated steatotic liver disease (MASLD), physical exercise improves insulin sensitivity and hepatic steatosis^[5]^. Yet, excessive exercise may impair mitochondrial function^[6]^ and inflammation^[7]^. Overtraining can also affect liver health by promoting hepatic fat accumulation^[8]^. Despite these observations, the molecular mechanisms underlying skeletal muscle-liver communication remain poorly understood.

The study from Liu *et al*. demonstrates that overtraining damages the liver by promoting inflammation and fibrosis^[9]^. In their Chinese population cohort, overtraining increases the levels of liver enzymes and fibrosis markers. In addition, overtraining in mice increases the production of lactate in the myocytes in skeletal muscles, which promotes the lactylation of vinexin, a protein coded by the sorbin and SH3 domain containing 3 (SORBS3) gene. *In vitro* studies showed that lactylated vinexin undergoes liquid-liquid phase separation (LLPS), facilitating its interaction with flotillin-1 and enabling the sorting of F-box protein 2 (FBXO2) into small extracellular vesicles (SEVs). These SEVs, termed “lactate bodies” by the authors, are released by myocytes and are taken up by the liver, where they induce hepatocyte apoptosis, subsequent hepatic stellate cell activation and liver fibrosis in mice. This work not only confirms previous observations that liver and skeletal muscles communicate via extracellular vesicles^[10]^, but also introduces the novel concept that skeletal muscle activity affects liver health, even in the absence of pre-existing liver disease. The study employs several mouse models, including genetic and pharmacological approaches. To demonstrate SEV trafficking from skeletal muscles to the liver in mice, the authors expressed cluster of differentiation 63 (CD63), a marker of multivesicular bodies, fused to green fluorescent protein (GFP) under the control of skeletal muscle gene myogenic factor 5 (*Myf5*) promoter. In both control and overtrained mouse groups, green fluorescence was detected in the liver, indicating a transfer of muscle-derived CD63^+^ particles to the liver. Validation of these findings in human cohorts would increase the relevance of the study. Another innovative aspect is the formation of condensates at the level of multivesicular bodies, the origin of a subpopulation of SEVs, through lactylated SORBS3 LLPS. The concept of condensates as a mechanism for selective cargo sorting into exosomes is relatively new^[11]^, and it has opened a path to a deeper understanding of exosome and SEV biology.

Although the authors do not have prior expertise in the field of skeletal muscle-liver crosstalk, this is a significant study that raises a few questions to be addressed in the future. While the paper describes how overtraining impacts liver injury in healthy mice, it would be of interest to understand whether overtraining worsens liver injury in mouse models of liver diseases, such as MASLD or alcohol-associated liver disease (ALD), which would be of high relevance for patients with liver disease. This leads to the need for developing tools to measure overtraining in patient populations and monitor their liver functions. Another aspect to be considered is the source of SEVs. While the examined SEVs were secreted from immortalized muscle cell lines such as C2C12, SEVs derived from primary skeletal muscle cells^[12]^ could provide a deeper understanding of how muscle communicates with the liver. In addition, the biology of the overtrained skeletal muscles could require further attention. Investigating proteomics and transcriptomics of the overtrained skeletal muscles might provide more insights into signals that can affect liver homeostasis and injury. Finally, sex-based biological differences have been reported to affect liver disease susceptibility^[13]^ and skeletal muscle mass and exercise^[14]^. Therefore, addressing sex differences would be a pertinent future direction.

In summary, by elucidating a mechanism of SEV-mediated inter-organ communication, the study by Liu *et al*. underpins the importance of the skeletal muscle-liver axis in healthy individuals and paves the way for studying it during liver disease^[9]^.

## DECLARATIONS

### Copyright

© The Author(s) 2025.
